# Case of Acute Rheumatism Ending in Paralysis

**Published:** 1806-01

**Authors:** 


### Case of Acute Rheumatism ending in Paralysis.
*Communicated by* Mr. Earnest.

My intention in publishing this case, is to show how sudden the transition is from one disease to another, and also to shew how necessary it is to arrest inflammatory complaints in the beginning. Had this patient applied for proper medical assistance when she was first attacked, it is very probable that this painful and lingering malady might have been prevented.

E. C. ætat. 27, a female, has been married, but is now a widow; she has never had more than one child, is low in stature, but is compact, muscular and strong, her complexion pale; she has in a general way enjoyed good health.

In the spring of the year 1803, after taking cold, she was attacked with acute rheumatism, which confined her many weeks, but at length she recovered and got quite well, and continued so until October 1804; when she was again seized with rheumatism, and the symptoms were, violent head-ache, rigors succeeded by heat and thirst, acute pains in the upper extremities, and in each shoulder. The bowels were costive, the arms swelled and inflamed, the pains in them were seldom long stationary, as sometimes the one would be attacked whilst the other remained free from pain, and in this manner both arms were alternately affected for four or five days; and all at once she lost the pain and swelling of the arms: but to this amendment there succeeded violent pain and fulness of the hypogastric region, accompanied with ischuria vesicalis; I say ischuria vesicalis, because there was a frequent propensity to pass water, without the power, and pain at the neck of the bladder; the urine that did pass off came by gradual distillation, and the fæces passed involuntarily; there was pain and weakness across the loins, and paralysis of the inferior extremities, with œdema; the pulse was quick, hard, and small; respiration short and quick; the appetite was very much impaired, and there was a loss of taste and smell. The catamenia had been very irregular for some time.

The patient had been afflicted in this distressing state for three weeks previous to her admission into the infirmary, which was on the 23d of November, when the following examination took place, viz. 1. The state of the uterus was inquired into, which proved to be in a natural and unimpregunimpregnated state. 2. The catheter was next introduced, which immediately discovered the cause of the pain and swelling of the abdomen, as five pints of strong fœtid urine were drawn off.* During this operation syncope came on, but the patient soon recovered again, when she declared herself free from the pain, weight, and fulness, which she so long had been troubled with. About two hours after emptying the bladder, a common enema was injected, but owing to the paralytic state of the sphincter ani and rectum, it was not retained one minute, neither did the injection bring away any fæces. A cardiac draught with tinct. opii was given at bed time.

Without particularizing any formula in the succeeding treatment, I shall briefly state the kinds of medicines exhibited in this singular case, viz. Internally, cantharides, scillæ, & camphor, bis indies. Elect. sennæ c. rheo pro re natâ. A generous diet. Externally, ut. pediluvio, o. n. applic. empl. cantharid. sacro, which were several times repeated. The abdomen and inferior extremities were regularly embrocated bis indies, with a stimulating liniment composed as follows: ℞. Camphor. dr. jß. æther vitriolic. ol. terebinth. rect. aa. dr. iv. ol. olivæ. unc. j. M. The patient persevered in these remedies from the 24th of November until the 24th of December, with very little advantage either to the bladder or rectum, as the stools still came away involuntarily, and the catheter was obliged to be regularly introduced two, and sometimes three times a day, for upwards of six weeks, before any natural effort was felt by the patient; and at each introduction there was never less than a pint of water taken off. The paralysis and œdema of the lower limbs, at this time, were much relieved by the use of the friction, &c. On the 24th of December, the cantharides et scillæ were omitted, in lieu of which, the following was prescribed. Mixtur. camphorat. spt. ammon. compt. tinct. guaiac. tinct. lavend. c. and other warm stomachics as occasion required. About a fortnight after the commencement of the last mentioned remedies, the patient appeared considerably better in every respect, and of course this indication of cure was ordered to be continued. She was kept in the Infirmary until the 5th of April, and at this time she

---

* The patient had not passed any water naturally, for nearly three weeks before, neither had she the power of making any efforts to advantage.

she was so far convalescent as to be able, by the aid of crutches, to walk about a little; she was therefore now admitted an out-patient, and her friends had directions given them to attend at the Infirmary on proper days for medicines, which she continued to take until May the 24th, 1805, when she walked herself to the Infirmary, (about a mile) and was discharged cured.

### Remarks.

The abdomen, in this case, becoming suddenly so much swelled and distended, in all probability, was the cause of compressing some of the most important nerves and blood-vessels, subservient to the principal functions of the lower half of the body, and thereby producing paralysis.

Dr. Cullen, in his First Lines of the Practice of Physic, asks the following question, viz. Can a palsy, occasioned by a compression, remain, though the compression be removed? And Dr. Rotheram, in a note, thus replies to the above question. "This question may be answered in the affirmative; because the structure of the nerve may be destroyed by the compression, and the nerve may therefore remain impervious to the nervous influence after the compression has been removed."

This kind of reasoning may be very just; but it appears somewhat ambiguous to me, as no length of time is mentioned as to the duration of compression being likely to render the nerves compressed impervious; and supposing that the paralysis in this case to have been occasioned either by tumour, effusion, or a plethoric state of the vessels themselves; it appears that an obstruction of this sort, for nearly six weeks together, had not the power of leaving the nerves impervious, or of depriving the parts affected wholly, either of sense or motion.

I have invariably remarked in practice, that acute rheumatism generally, if not always, yields to the following remedies, viz. Bleeding freely from the arm, and repeating the operation according to the urgency of the symptoms, by evacuating the stomach and bowels with the antimonium tartarisatum, and afterwards a proper and continued course of diaphoretics, calomel, and magnesia vitriolata pro re nata, cinchona of course follows at a proper time. The regimen should be diluent drinks and an attenuating diet.*

I think

* Opiates in these cases are often necessary, and the patients suffering under this painful malady generally experience the most happy and soothing effects from full doses (℈j ad ℥ß) of the pulv. ipecac. comp. in the form of a bolus.

I think I may venture to add, that if this patient had been treated in the way just mentioned, when she was first attacked with rheumatism, that the paralysis would in all probability have been prevented.

*December* 7, 1805.

